# Lesion-based detection of early chemosensitivity using serial static FDG PET/CT in metastatic colorectal cancer

**DOI:** 10.1007/s00259-012-2172-2

**Published:** 2012-06-19

**Authors:** Irène Buvat, Hatem Necib, Camilo Garcia, Antoine Wagner, Bruno Vanderlinden, Patrick Emonts, Alain Hendlisz, Patrick Flamen

**Affiliations:** 1 IMNC UMR 8165 CNRS – Paris 7 and Paris 11 Universities, Campus d’Orsay, Bâtiment 440, 91406 Orsay cedex, France; 2 Nuclear Medicine Department, Institut Jules Bordet, Université Libre de Bruxelles, Brussels, Belgium; 3 Radiology Department, Institut Jules Bordet, Université Libre de Bruxelles, Brussels, Belgium; 4 Digestive Oncology, Institut Jules Bordet, Université Libre de Bruxelles, Brussels, Belgium

**Keywords:** PET/CT, Objective response prediction, FDG, Advanced colorectal cancer, EORTC criteria, SUV

## Abstract

**Purpose:**

Medical oncology needs early identification of patients that are not responding to systemic therapy. ^18^F-Fluorodeoxyglucose (FDG) positron emission tomography (PET) performed before and early during treatment has been proposed for this purpose. However, the best way to assess the change in FDG uptake between two scans has not been identified. We studied cutoff thresholds to identify responding tumours as a function of the method used to measure tumour uptake.

**Methods:**

The study included 28 metastatic colorectal cancer (mCRC) patients who underwent 2 FDG PET/CT scans (baseline and at day 14 of the first course of polychemotherapy). For 78 tumour lesions, 4 standardized uptake value (SUV) indices were measured: maximum SUV (SUV_max_) and mean SUV in a region obtained using an isocontour (SUV_40 %_), with each of these SUV normalized either by the patient body weight (BW) or body surface area (BSA). The per cent change and absolute change in tumour uptake between the baseline and the early PET scans were measured based on these four indices. These changes were correlated to the RECIST 1.0-based response using contrast-enhanced CT at baseline and at 6–8 weeks on treatment.

**Results:**

The 78 tumours were classified as non-responding (NRL, *n* = 58) and responding lesions (RL, *n* = 20). Receiver-operating characteristic (ROC) curves characterizing the performance in NRL/RL classification using early FDG PET uptake had areas under the curve between 0.75 and 0.84, without significant difference between the indices. The cutoff threshold in FDG uptake per cent change to get a 95 % sensitivity of RL detection depended on the way uptake was measured: −14 % (specificity of 53 %) and −22 % (specificity of 64 %) for SUV_max_ and SUV_40 %_, respectively. Thresholds expressed as absolute SUV decrease instead of per cent change were less sensitive to the SUV definition: an SUV decline by 1.2 yielded a sensitivity of RL detection of 95 % for SUV_max_ and SUV_40 %_. For a given cutoff threshold, the sensitivity was the same whatever the normalization (by BSA or BW).

**Conclusion:**

A 14 % drop of tumour FDG SUV_max_, 22 % drop of SUV_40 %_ or 1.2 drop of SUV_max_ or SUV_mean_ after one single course of polychemotherapy predicts objective response in mCRC lesions with a high sensitivity, potentially allowing the early identification of non-responding patients.

## Introduction

^18^F-fluorodeoxyglucose (FDG) positron emission tomography (PET) was proposed for the early assessment of treatment response in cancer patients almost 20 years ago [1, 2]. Since then, the potential of FDG PET for cancer patient monitoring has been well established [3]. In that context, the usefulness of quantitation of FDG uptake in tumours compared to a visual analysis only has also been suggested [4, 5]. Yet, there is currently no validation of the best method to be used to measure FDG uptake in tumours and the cutoff thresholds of decline in uptake to consider for identifying responding tumours [6–8]. Practical considerations have led to a widespread use of the standardized uptake value (SUV) as a metric for characterizing tumour uptake. However, many methods can be used to estimate SUV, mostly varying in the way SUV is normalized (to body mass, lean body mass or body surface area) and in the tumour region of interest (ROI) used to measure SUV (single voxel, fixed number of voxels, tumour-dependent number of voxels chosen either manually or semiautomatically). It is now obvious that standardizing quantitative procedures and quality control is required to make the most of ^18^FDG PET in the context of therapy monitoring [9, 10]. As early as 1999, the European Organization for Research and Treatment of Cancer (EORTC) published recommendations on the measurement of tumour response using ^18^FDG PET, based on the use of SUV [11]. These recommendations were derived from an extensive review of the limited data available in the literature at that time. The recommended approach has been used in a large number of studies since then. However, as the recommendations did not include any strict description of the way SUV had to be measured, the so-called EORTC 1999 criteria most often only refer to the cutoff thresholds to be used for distinguishing between progressive metabolic disease (PMD), stable metabolic disease (SMD), partial metabolic response (PMR) and complete metabolic response (CMR). The aim of our study was to investigate the relevance of the recommended cutoff thresholds as a function of the method used to estimate the SUV using a set of 28 patients with metastatic colorectal cancer.

## Materials and methods

### Patients

Twenty-eight patients (mean age 62.8 years, range 23–83 years) with metastatic colorectal cancer treated at the Institut Jules Bordet, Brussels, Belgium, were included in the study. All patients were prospectively recruited, as part of a prospective clinical trial on a larger cohort of patients for assessing the role of early FDG PET/CT as a predictor of the RECIST-based morphological response to chemotherapy in metastatic colorectal cancer patients [12]. The study was approved by the Ethics Committee of the Institut Bordet and registered in clinicaltrials.gov under number NCT00741481. Written informed consent was obtained from all patients. All patients presented advanced metastatic colorectal cancer deemed to be treated by chemotherapy. Chemotherapy involved FOLFOX (19 patients), FOLFIRI (9 patients) and capecitabine (1 patient) as first- (20) or second-line (9) treatments. The mean number of lesions per patient was 3 (range 1–8). A total of 78 lesions were analysed (3 primary, 49 in the liver, 8 in the lungs, 10 in the peritoneum and 8 at other various locations).

### Imaging protocols

Each patient had a first helical diagnostic CT with or without IV contrast injection (depending on the evaluated lesion) 8 days (range 0–23 days) on average before the first FDG PET/CT, and a second CT scan after 5–8 weeks on therapy, or sooner in case of clinical suspicion of progression. Axial slice thickness was 3 or 5 mm, depending on the scanner used for the CT.

Each patient also underwent a baseline FDG PET/CT scan just before the start of chemotherapy (day 0) and a follow-up scan at day 14 (mean ± 1 SD = 13.7 ± 2.7). Patient preparation, imaging and reconstruction protocols were kept constant for serial scans in the same patient. The FDG PET/CT images were obtained using a GE Discovery LS system, 60 min (range 53–87 min) after injection of 4 MBq/kg. For all patients included in the study, the post-injection time between the baseline and early PET did not differ by more than 15 min. PET images were reconstructed with the built-in GE Advance software, using the ordered subset expectation maximization (OSEM) algorithm with 2 iterations and 28 subsets, and a 5.45 mm full-width at half-maximum (FWHM) Gaussian post-filtering. The images were corrected for attenuation based on the CT and for scatter. The CT was performed with a 4-slice multidetector helical scanner (Lightspeed, GE Medical Systems). The tension was 120 kV, and the range of currents was set from 30 to 200 mA. The actual current within this range was determined by the AutomA, an algorithm from GE that modifies the intensity during the acquisition depending on a noise index and the attenuation information given by the scout view. The noise index used for this low-dose CT was 25. The other parameters were 0.5 s per CT rotation, a pitch of 1.5 and a table speed of 15 mm/rotation. The matrix of CT images was 512 × 512 (0.98 × 0.98 mm pixel size) with a 5-mm slice thickness. The PET volumes (128 × 128 pixels of 3.91 × 3.91 mm, 4.25-mm slice thickness) and the CT volumes were systematically coregistered using the GE software (LightSpeedAppsct_dst_dls_1.7_R2.9N.IRIX646.5).

### CT image interpretation

Target lesions were initially identified by a senior radiologist and two nuclear medicine physicians in a joint reading session. Only FDG-avid lesions clearly individualized on both baseline PET and diagnostic CT and that had a minimal diameter of 15 mm on the baseline diagnostic CT were considered as target lesions. A lesion-by-lesion analysis was performed. CT was interpreted according to RECIST criteria 1.0, as follows [13]:

- Complete response was defined as the disappearance of the target lesion with no evidence of tumour elsewhere.
- Partial response was defined as at least a 30 % reduction in the tumour dimension, where the tumour dimension was defined as the single longest dimension of the tumour in the transaxial plane.
- Progressive lesion was defined as an increase of at least 20 % in the tumour dimension.
- A stable lesion was a lesion that was visible but did not meet the partial response or progressive lesion criteria. For these stable lesions, confirmation of stable disease status was obtained by an additional CT scan (i.e. a third CT scan) after 6–8 more weeks.

Complete response and partial response were considered as responding lesions (RL), while progressive lesions and stable lesions were considered as non-responding lesions (NRL).

### PET image quantitation

To compare PET scans acquired before and during therapy, the images were converted in SUV units. Two normalizations were considered to derive the SUV images:

- Normalization by the injected activity corrected for radioactive decay and divided by the body surface area (BSA). The BSA (m^2^) was given by BSA = 0.00718 × W^0.425^ × H^0.715^, where *W* is the patient weight (kg) and *H* is the patient’s height (cm), as recommended by the EORTC [11, 14].
- Normalization by the injected activity corrected for radioactive decay and divided by the patient’s body weight (BW), as most commonly used in clinical practice.

For each patient, each target tumour and each SUV normalization, two SUV indices frequently used in clinical practice were measured in each of the two PET scans available for the patient (baseline and during therapy):

- Maximum SUV (SUV_max_) in the tumour, corresponding to a 1-voxel ROI.
- Mean SUV in the tumour in a 3-D ROI obtained using an isocontour set at 40 % of the SUV_max_ in the tumour (SUV_40 %_). This region and the number of voxels it included could thus be different for the baseline and early PET scans.

From these measurements, eight indices characterizing the change in tumour uptake between the baseline and the early PET scans were derived. Four indices, namely ΔSUV_max_BSA_, ΔSUV_40 %_BSA_, ΔSUV_max_BW_ and ΔSUV_40 %_BW_, corresponded to per cent changes in SUV between the baseline and early PET scans, with:

$$ \Delta {\text{SU}}{{\text{V}}_{{{\text{X}}\_ {\text{Y}}}}} = 100 \times \left( {{\text{SU}}{{\text{V}}_{{{\text{X}}\_ {\text{Y}}}}}^{{{\text{early}}}} - {\text{ SU}}{{\text{V}}_{{{\text{X}}\_ {\text{Y}}}}}^{{{\text{baseline}}}}} \right)/{\text{SU}}{{\text{V}}_{{{\text{X}}\_ {\text{Y}}}}}^{{{\text{baseline}}}} $$

*X* corresponded to the two SUV measurement methods (max or 40 %) and *Y* corresponded to the two normalization methods (BSA or BW).

Four indices, diffSUV_max_BW_, diffSUV_40 %_BW_, diffSUV_max_BSA_ and diffSUV_40 %_BSA_, measured the absolute change in SUV between the baseline and early PET scans, with:

$$ {\text{diffSU}}{{\text{V}}_{{{\text{X}}\_ {\text{Y}}}}} = {\text{SU}}{{\text{V}}_{{{\text{X}}\_ {\text{Y}}}}}^{{{\text{early}}}} - {\text{SU}}{{\text{V}}_{{{\text{X}}\_ {\text{Y}}}}}^{{{\text{baseline}}}} $$

### Receiver-operating characteristic curve analysis

To determine how well the eight indices characterizing the change in tumour uptake could distinguish between RL and NRL, nonparametric receiver-operating characteristic (ROC) curve analysis was performed using the ROCKIT software (http://radiology.uchicago.edu/?q=MetzROCsoftware). By considering the RL and NRL classification given by the RECIST criteria, ROC curves were plotted for ΔSUV_max_BSA_, ΔSUV_40 %_BSA_, ΔSUV_max_BW_, ΔSUV_40 %_BW_, diffSUV_max_BW_, diffSUV_40 %_BW_, diffSUV_max_BSA_ and diffSUV_40 %_BSA_, by varying a cutoff threshold T: if ΔSUV_X_Y_ or diffSUV_X_Y_ was less than T, the tumour was classified as RL, while if ΔSUV_X_Y_ or diffSUV_X_Y_ was equal to or greater than T, it was classified as NRL. The ROC curves were obtained by plotting the sensitivity of detecting RL as a function of [1 minus the specificity].

The cutoff thresholds corresponding to a 95 % sensitivity of detecting RL were deduced from the ROC curves of each ΔSUV_X_Y or_ or diffSUV_X_Y_ index, and the corresponding specificity values were determined. The thresholds yielding a negative predictive value (NPV) of 95 % were also determined for each ΔSUV_X_Y_ and diffSUV_X_Y_ index and the corresponding positive predictive values (PPV) were deduced.

## Results

### Tumour classification

According to RECIST, the 78 tumours were classified as 20 partial responses, 44 stable lesions and 14 progressive lesions. This corresponds to 20 RL and 58 NRL.

### Performance of the eight indices to identify RL

**Fig. 1:**
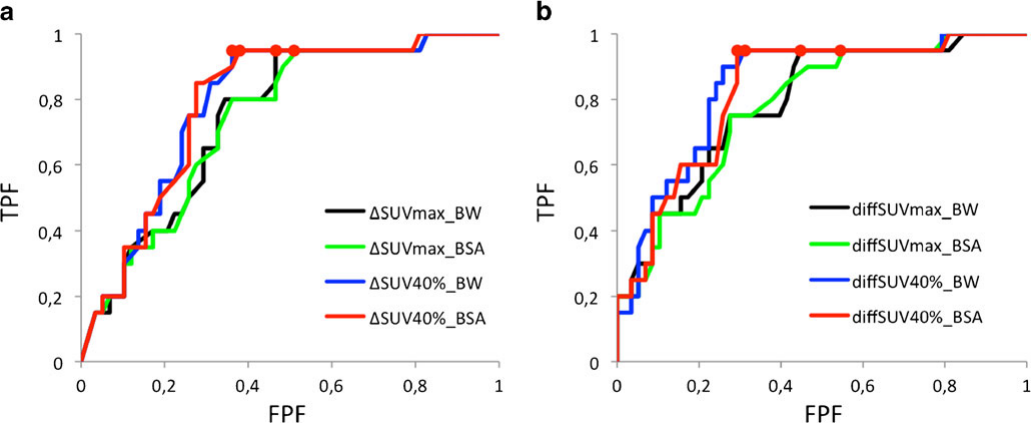
**a** ROC curves corresponding to the ΔSUV_max_BSA_, ΔSUV_40 %_BSA_, ΔSUV_max_BW_ and ΔSUV_40 %_BW_ indices used to discriminate between RL and NRL. **b** ROC curves corresponding to the diffSUV_max_BSA_, diffSUV_40 %_BSA_, diffSUV_max_BW_ and diff_SUV40 %_BW_ indices used to discriminate between RL and NRL. *Red points* show the operating points for which a 95 % sensitivity is achieved with the maximum specificity

Figure 1 shows the eight ROC curves representing the performance of the eight indices to distinguish between RL and NRL when considering all lesions. The areas under the curves (AUC) are summarized in Table 1. None of the AUC was significantly different from the others (nonparametric paired tests with α = 0.05) [15].

**Table 1:** Areas under the ROC curves (±1 standard deviation) as a function of the index used to discriminate RL and NRL when considering all 78 lesions

	max_BW	40 %_BW	max_BSA	40 %_BSA
ΔSUV_X_Y_	0.75 ± 0.06	0.79 ± 0.06	0.74 ± 0.06	0.79 ± 0.06
SUV_X_Y_^early^–SUV_X_Y_^baseline^	0.79 ± 0.06	0.84 ± 0.05	0.78 ± 0.06	0.83 ± 0.05

### Cutoff thresholds of FDG uptake change for identifying RL

Table 2 gives the cutoff thresholds and corresponding sensitivity, specificity, accuracy, NPV and PPV for the different indices. Four sets of results are shown: those corresponding to setting the cutoff threshold to 15 or 25 % as recommended by the EORTC as well as the results obtained when aiming at a 95 % sensitivity for detecting RL or when aiming at a 95 % NPV. In these latter cases, values for the four indices normalized by BSA are not included as they always differ by less than 2 % from the corresponding indices normalized by BW.

**Table 2:** Cutoff threshold and corresponding sensitivity, specificity, NPV and PPV for detecting RL using FDG PET. In total, there were 20 RL and 58 NRL

Index	Threshold (number rated NRL-number rated RL)	Sensitivity	Specificity	NPV	PPV	Accuracy
Using cutoff threshold of 15 %						
ΔSUV_max_BW_	15 % (35-43)	80 %	53 %	89 %	37 %	60 %
ΔSUV_40 %_BW_	15 % (32-46)	95 %	53 %	97 %	41 %	64 %
ΔSUV_max_BSA_	15 % (35-43)	80 %	53 %	89 %	37 %	60 %
ΔSUV_40 %_BSA_	15 % (33-45)	95 %	55 %	97 %	42 %	65 %
Using cutoff threshold of 25 %						
ΔSUV_max_BW_	25 % (46-32)	65 %	67 %	85 %	41 %	67 %
ΔSUV_40 %_BW_	25 % (39-39)	90 %	64 %	95 %	46 %	71 %
ΔSUV_max_BSA_	25 % (46-32)	65 %	67 %	85 %	41 %	67 %
ΔSUV_40 %_BSA_	25 % (39-39)	90 %	64 %	95 %	46 %	71 %
Aiming at a 95 % sensitivity of detection of RL						
ΔSUV_max_BW_	14 % (32-46)	95 %	53 %	97 %	41 %	64 %
ΔSUV_40 %_BW_	22 % (38-40)	95 %	64 %	97 %	47 %	72 %
diffSUV_max_BW_	1.4 (33-45)	95 %	55 %	97 %	42 %	65 %
diffSUV_40 %_BW_	1.2 (41-37)	95 %	69 %	98 %	51 %	76 %
Aiming at a 95 % NPV for detection of RL						
ΔSUV_max_BW_	14 % (32-46)	95 %	53 %	97 %	41 %	64 %
ΔSUV_40 %_BW_	22 % (38-40)	95 %	64 %	97 %	47 %	72 %
diffSUV_max_BW_	1.4 (33-45)	95 %	55 %	97 %	42 %	65 %
diffSUV_40 %_BW_	1.2 (41-37)	95 %	69 %	98 %	51 %	76 %

Table 3 shows the 2 × 2 arrays describing the agreement between SUV_40 %_BW_ and SUV_max_BW_, when setting the cutoff threshold to 15 % (left array) and 25 % (right array). The number of disagreements was about the same whatever the cutoff threshold (18/78 for a threshold set to 15 % and 17/78 for a threshold set to 25 %).

**Table 3:** Agreement/discrepancies between the SUV_max_BW_ and SUV_40 %_BW_ classification

Cutoff threshold = 15 %			
		SUV_40 %_BW_	
		RL	NRL
SUV_max_BW_	RL	36	7
	NRL	11	24
Cutoff threshold = 25 %			
		SUV_40 %_BW_	
		RL	NRL
SUV_max_BW_	RL	27	5
	NRL	12	34

## Discussion

We tested the relevance of the cutoff thresholds of treatment-induced FDG uptake change to characterize the early tumour response to (poly)chemotherapy on a lesion-by-lesion basis in a prospective cohort of metastatic colorectal cancer patients. The oncological rationale behind early response assessment is the timely identification of NRL and/or patients so that inappropriate treatments can be rapidly replaced or adjusted. Doing this the highest care should be provided in order not to falsely exclude patients from a potentially beneficial treatment modality. Therefore, our testing aimed at 95 % sensitivity and NPV for prediction of response.

To perform such an investigation, we needed to have an FDG PET-independent way to distinguish RL and NRL. In our protocol, the objective lesion size-based tumour response according to RECIST, derived from follow-up CT scans performed at 6–8 weeks on therapy, was used as the reference of response. As a result, our study actually investigated on a lesion-by-lesion basis how appropriate FDG PET-based metabolic criteria were for predicting the size-based tumour response later seen using the CT scan. We also investigated whether the thresholds to be used depended on the way SUV was calculated.

Our results (Fig. 1 and Table 1) first suggest that all SUV indices considered in this study yielded similar performance of RL/NRL classification, since the ROC curves were similar with non-significant differences between the AUC. This means that neither the normalization used for calculating the SUV (BSA or BW), nor the way uptake was measured (using SUV_max_ or SUV_40 %_) nor the way change in uptake was evaluated (per cent change or absolute change) did impact the classification performance. FDG PET images therefore include robust quantitative information for identifying RL. The index that most accurately reflects such information still needs to be identified, and our results suggest that SUV_max_ and SUV_mean_ are two candidates that performed well in our context. However, our results also show that the cutoff threshold yielding a 95 % sensitivity for detecting RL is substantially different when using SUV_max_ (−14 %) or SUV_mean_ (−21 or −22 % depending on the SUV normalization) (Table 2). All these values are consistent with the range (−25 to −15 %) that the EORTC recommended when assessing the treatment response after a single cycle of chemotherapy. Yet, an important finding is that using a given cutoff threshold the sensitivity of detecting RL depends on the way SUV is measured. For instance, considering the lower bound of the range recommended by the EORTC (15 % decline in SUV for establishing RL), sensitivity of detecting RL is 95 % with ΔSUV_40 %_BW_ (for a 53 % specificity, NPV of 97 %) but only 80 % with ΔSUV_max_BW_ (for a 53 % specificity, NPV of 89 %). Considering the upper range recommended by the EORTC (25 % decline in SUV for establishing RL), sensitivity of detecting RL is 90 % with ΔSUV_40 %_BW_ (for a 64 % specificity, NPV of 95 %) but only 65 % with ΔSUV_max_BW_ (for a 67 % specificity, NPV of 85 %). Considering cutoff thresholds between 15 and 25 % for any ΔSUV_X_Y_ index always yielded an NPV equal to or greater than 85 %, which confirms the relevance of the EORTC criteria for assessing tumour response. Yet, using SUV_mean_, higher cutoff values should be used to yield NPV similar to those obtained with SUV_max_. Depending on the context of patient management, the target sensitivity, specificity, NPV or PPV may differ from the one considered in our work. Yet, based on our results, it is expected that the threshold to be used to reach the target values will depend on the way the SUV is measured. In this work, we only studied SUV_max_ and SUV_40 %_, as they are frequently used in routine daily practice, but it is likely that other thresholds would be found “optimal” (in terms of targeting a sensitivity or NPV values) if other SUV definitions were used.

Although change is SUV is most often evaluated in terms of per cent decline, we also studied whether analysing absolute change in SUV units instead of per cent change could improve the identification of RL and NRL. Figure 1 and Table 1 suggest that absolute change and per cent change convey about the same information for lesion classification: the AUC obtained with the per cent change were not significantly different from the AUC obtained with the absolute change for a given X_Y combination (e.g. ΔSUV_max_BW_ against diffSUV_max_BW_). The cutoff threshold required to reach a 95 % sensitivity of detecting RL was 1.4 (decrease of SUV by at least 1.4, Table 2) with SUV_max_BW_ (specificity of 55 %, NPV of 97 %) and was 1.2 with SUV_40 %_BW_ (specificity of 69 %, NPV of 98 %). Using a threshold of 1.2 with SUV_max_BW_ leaves the sensitivity and NPV almost unchanged (sensitivity of 95 % and NPV of 96 %), but decreased the specificity (40 %) and overall accuracy (54 %). Considering a threshold of 1.4 with SUV_40 %_BW_, the sensitivity would be 85 % only (specificity of 74 %, NPV of 93 %). The cutoff threshold is actually less dependent on the way SUV is measured when expressed as an absolute decrease in SUV (varying from 1.2 to 1.4 decline to reach a sensitivity of 95 %, depending on whether one measures SUV_40 %_BW_ or SUV_max_BW_) than when expressed as a per cent change (varying between 14 and 22 % decline). Further investigations including other tumour types and imaging protocols are needed to determine whether relying on absolute decrease of SUV instead of per cent decrease could make the threshold less dependent on the way SUV is measured. Table 2 also demonstrates that when setting the sensitivity or NPV to 95 %, diffSUV_40 %_BW_ had the highest accuracy compared with all other measured changes.

Table 2 shows that the cutoff threshold did not depend on the way SUV was normalized. This lack of difference is due to the very high correlation between the ΔSUV_X_BW_ and ΔSUV_X_BSA_ values (*r* ^2^ > 0.99) or between diffSUV_X_BW_ and diffSUV_X_BSA_ (*r* ^2^ > 0.98). As a result, when using early PET for patient monitoring, SUV can be calculated by normalizing either by the BSA or by the BW. Although EORTC recommended the use of BSA for normalization, most centres actually use BW for convenience. Our results suggest that this does not make a difference and that the thresholds recommended by the EORTC when considering BSA as a normalizing factor are still valid when using BW instead. This conclusion is only valid in case of early treatment response evaluations, which are often performed at one course of chemotherapy before major changes in body tissue composition occur (i.e. loss of the non-FDG-avid fat compartment).

It has been suggested that “the 15 % decline in SUV in the original EORTC criteria for early response is probably too modest to reliably be discerned from variability in the study and likely is insufficient to be medically relevant based on data developed since that time” [8]. Our results did not confirm this statement and demonstrated that using a 14 % decline in SUV_max_ was appropriate to identify RL with a sensitivity of 95 %.

One of the major observations of the study was that when applying the optimal cutoffs for treatment response in terms of sensitivity (aiming at 95 %) the specificities and positive predictive values were low (ranging from 53 to 69 % and 41 to 51 %, respectively). The understanding of this is that an early metabolic response does not always translate to a significant reduction of size later on (i.e. 30 % according to RECIST). This could indicate that in some lesions the metabolic response is transient and short lasting or that the size criteria which are used as the reference in this study are not sensitive enough to detect response. Differentiation of these would need a true reference of response, based on tissue analysis of one or more target lesions via biopsy, which was not performed in the current study.

Our results were obtained for a specific cohort of patients and monitoring protocol. Given that in our patients most lesions were liver metastases (49 of 78 lesions), all analyses were also systematically performed by including only the 49 liver metastases (16 RL and 33 NRL according to RECIST 1.0) (results not shown). No significant differences were found compared to the results obtained when including all lesions, and all conclusions drawn from the studies including all lesions were identical when restricting the analysis to the liver metastases. Whether the recommended thresholds are also valid for other types of tumours, other treatment modalities and other monitoring protocols remains to be investigated. The translation of the current lesion-by-lesion analysis data into a patient-based response classification using treatment outcome measures such as time to progression and/or overall survival has been recently reported by Hendlisz et al. [16].

In this paper, early response was evaluated using acquisition and analysis protocols frequently used in nuclear medicine departments. Yet, more sophisticated protocols or analysis methods are also being investigated for the evaluation of early response, including the use of dynamic protocols allowing for calculation of kinetic parameters, or of parametric imaging methods (e.g. [17–19]). Such approaches show promise for improved assessment of early response and might outperform more conventional data analysis.

### Conclusion

Using a prospective cohort of 28 patients with metastatic colorectal cancer (78 target lesions in total), we showed that the SUV cutoff thresholds recommended by the EORTC to identify tumours responding to therapy yielded high sensitivity and NPV for detecting tumour response after a single course of polychemotherapy. However, the sensitivity obtained when using the recommended 15 % decrease in SUV to identify a tumour response after one cycle of chemotherapy varied from 80 % for ΔSUV_max_ (specificity of 53 %, NPV of 89 %) to 95 % for ΔSUV_40 %_ (specificity of 53 %, NPV of 97 %). Defining the threshold as an absolute SUV decrease instead of a per cent change made the threshold less sensitive to the SUV definition: an SUV decline by 1.2 yielded a sensitivity of 95 % for SUV_max_ and SUV_40 %_. For a given cutoff threshold, the sensitivity was the same regardless of whether SUV was normalized by BSA or by BW.
